# A rare case of hereditary multiple osteochondromas

**DOI:** 10.11604/pamj.2020.37.173.25960

**Published:** 2020-10-21

**Authors:** Rakesh Khatana, Renu Rathi

**Affiliations:** 1 Mahatma Gandhi Ayurved College Hospital and Research Centre, Datta Meghe Institute of Medical Sciences, Wardha, Maharashtra, India

**Keywords:** Osteochondromas, hereditary, osteocartilaginous

## Image in medicine

Five (5) years old male patient came to outpatient department with complain of multiple swelling over chest region with upper extremities. The clinical examination revealed that there is chest multiple mass growth of about 3-4 cm bilaterally each over chest, upper extremities and forehead. The development of multiple benign osteocartilaginous masses (exostoses) begins with the relation with the ends of long bones of the lower limbs such as the femurs and tibias and of the upper limbs such as the humerus and forearm bones with chest region. The patient also had complains of difficulty in eating, finger grips, as well as loss of functional independence. On clinical examination the patient was diagnosed with a rare case of hereditary multiple osteochondromas (HMO).

**Figure 1:**
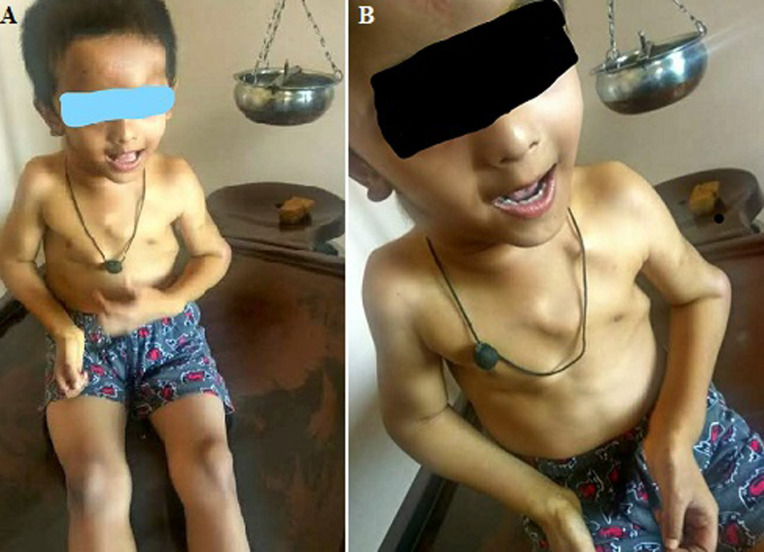
A) anterior view of the patient; B) multiple osteochondromas showing in the chest region

